# Effect of Bladder Injections of Botulinum Neurotoxin A on Biomarkers Associated with Inflammation and Urinary Infections in Patients with Neurogenic Detrusor Overactivity-Associated Incontinence: A Pilot, Prospective, Human Study

**DOI:** 10.3390/ijms27021110

**Published:** 2026-01-22

**Authors:** Sotirios Gatsos, Elena Constantinou, Dimitrios Koutsoumparis, Michael Samarinas, Konstantinos Drosos, Maria Papaioannou, Andigoni Malousi, Eudoxia G. Hatzivassiliou, Apostolos Apostolidis

**Affiliations:** 1 2nd Department of Urology, Aristotle University of Thessaloniki, ‘Papageorgiou’ General Hospital, Ring Road, Nea Efkarpia, 56403 Thessaloniki, Greece; sot.gatsos@gmail.com (S.G.); msamaria@auth.gr (M.S.); kodrossos@hotmail.com (K.D.); 2 Department of Biological Chemistry, School of Medicine, Aristotle University of Thessaloniki, 54124 Thessaloniki, Greece; ekonstantinou@auth.gr (E.C.); dimitriskou92@gmail.com (D.K.); mpapaioannou@auth.gr (M.P.); andigoni@auth.gr (A.M.)

**Keywords:** botulinum toxin, overactive bladder, detrusor overactivity, inflammation, urinary tract infections, biomarkers, prostaglandin, Toll-like receptors

## Abstract

Conflicting data exist regarding the effect of intradetrusor BoNT/A on the incidence of urinary tract infections (UTIs) in patients with neurogenic detrusor overactivity (NDO), contrary to the increase in UTIs noted in patients with idiopathic OAB. Associations between UTIs, chronic inflammation, and bladder overactivity are acknowledged, albeit not fully understood. Chronic bladder inflammation is common in both NDO and OAB patients, and both animal and human studies suggest a beneficial effect of BoNT/A on both urinary and systemic levels of inflammatory markers. To explore whether intradetrusor BoNT/A injections affect the background for the incidence of UTIs in humans, we investigated in parallel the effect of intradetrusor BoNT/A on the incidence of UTIs and on the urine mRNA levels of urinary pathogen-detecting Toll-like receptors TLR2, TLR4, and TLR5 and of factors acting as intermediates of immune response and promoters of inflammatory reactions (IL1β, IL6, TNFα, and PGE_2_). For this purpose, we recruited 22 patients with NDO-associated incontinence who received at least one bladder BoNT/A injection. Urine specimens for the study of UTIs were obtained before the procedure and at routine urodynamic follow-ups at 4–6 weeks, 6 and 12 months post-BoNT/A, and at clinical relapse, while urine specimens for the study of biomarkers were collected at the time of BoNT/A injection and at the abovementioned follow-ups thereafter. Urine specimens from 10 adult healthy volunteers with no OAB symptoms served as the control group in the biomarker study. The genes of interest in the urine were studied by RNA isolation, reverse transcription, and real-time PCR. The urine mRNAs of all biomarkers tested appeared to be upregulated in the patients’ samples compared with the controls, albeit only TLR2 and TLR5 mRNA increases were statistically significant. A progressive downregulation of TLR2, TLR5, IL1β, and IL6 urine mRNAs was noted at one and six months post-BoNT/A. TNFα and PGE_2_ mRNAs showed a transient increase at one month post-BoNT/A followed by a dramatic drop at the six months’ follow-up. A similar trend for progressive decline was also noticed in the prevalence of both positive urine cultures and symptomatic UTIs in the same timepoints and additionally at 12 months post-treatment in patients who still benefited from the BoNT/A treatment. Upon clinical relapse, the mRNA levels of PGE_2_, IL1β, and IL6 increased in parallel with an increase in the prevalence of UTIs, while the levels of TLRs and TNF-α did not follow the same trend. In summary, intradetrusor BoNT/A injections achieved significant decreases in the urine mRNA levels of pathogen-detecting TLRs, immune response, and inflammation mediator cytokines and PGE_2_ in our cohort of patients with NDO-associated incontinence. In parallel, decreases were noted in both the incidence of symptomatic UTIs and rates of positive urine cultures. At the time of clinical relapse, the markers of inflammation and immune response, but not TLRs, were upregulated in parallel with the increased incidence of UTIs, suggesting that the studied genes PGE_2_, IL1β, and IL6 could be further explored as potential biomarkers for inflammation/immune response and UTIs in the neurogenic population.

## 1. Introduction

Overactive bladder (OAB) is a very prevalent condition, affecting between 1 in 10 and 1 in 3 people in various epidemiological studies [1]. Although the clinical manifestation of the syndrome is clearly defined, namely, a syndrome characterized by urinary urgency, usually with urinary frequency and nocturia, with or without urgency urinary incontinence, the underlying pathophysiology is multifaceted and a subject of ongoing research [2]. Inflammation is an emerging factor in the pathogenesis of OAB, with mounting evidence supporting its role [3]. Patients with OAB have been found to have increased levels of urine biomarkers associated with inflammation (such as TNF-a, IL-2, NGF), as well as serum/systemic biomarkers [4,5]. Additionally, urinary tract infections (UTIs) are implicated in the pathogenesis of a subgroup of patients, with a bidirectional connection of OAB and UTIs, as patients with OAB have a higher risk of developing infections [6,7].

Intradetrusor injection of botulinum toxin A (BoNT/A) is an established third-line treatment for idiopathic OAB and neurogenic detrusor overactivity (NDO)-associated incontinence refractory to oral pharmacotherapy. BoNT/A was known to act via inhibition of acetylcholine release at the level of the neuromuscular junction [8], but human and animal bladder studies support an additional inhibiting effect on peripheral sensory and central neural pathways associated with bladder control [9]. Beyond the neuromodulatory properties, BoNT/A has demonstrated potential anti-inflammatory action. Studies in rats have reported inhibition of the release of sensory neurotransmitters in a setting of induced acute and chronic bladder inflammation, signifying a potential therapeutic role [10]. Regarding BoNT/A’s effect on the incidence of UTIs, an increase in UTIs after the treatment is well documented by many patient cohorts of idiopathic OAB, with the incidence being around 20% [11,12]. The data are contradictory for patients with NDO, as BoNT/A has been demonstrated to reduce the incidence of symptomatic UTIs in observational studies but not in randomized controlled trials [13]. Nevertheless, the exact effect of BoNT/A on human urinary bladder inflammation remains to be elucidated.

This pilot prospective study aims to explore whether intradetrusor BoNT/A injections affect the background for the incidence of UTIs by evaluating the effect of BoNT/A bladder injections on biomarkers associated with inflammation and urinary infections in the urine of patients with OAB and in parallel with the effect of BoNT/A on the incidence of UTIs. Toll-like receptors (TLRs) are primary sensors for pathogen-associated molecular pathways [14]. Therefore, we studied the mRNA levels of TLR2, TLR4, and TLR5 in the urine of patients before and after intradetrusor BoNT/A injections, alongside the expression of inflammatory cytokines, IL1b, IL6, TNFα, and prostaglandin E2 (PGE_2_).

## 2. Results

### 2.1. Urinary Tract Infection Study

A total of 22 patients (14 women, 8 men) were enrolled and completed the first intradetrusor injection of BoNT/A. The majority (n = 11) suffered from multiple sclerosis, n = 7 from spinal cord injury, n = 2 from spina bifida, one from Parkinson’s disease, and one from diabetes. Mean age was 46.8 years (age range 14–79 years) and disease duration exceeded one year in all cases. Follow-up data were complete for all 22 participants at 1 month, for 14 patients (63.6%) at 6 months, and for 6 patients (27.3%) at 12 months after the first treatment. Six patients subsequently received a second BoNT/A injection; all six were evaluated at 1 month, and three had follow-up data at 6 months after the second injection.

All study participants were considered complete responders to treatment, using zero or one safety pad per day, at least up to 6 months post-injection. Significant improvements were recorded in MCC (pre-first BoNT/A 333.9 ± 208.3 mL vs. 678.8 ± 415.6 mL at 1 month [*p* = 0.000], 658.6 ± 241.7 mL at 6 months [*p* = 0.000], 560.7 ± 271.7 mL at 12 months [*p* = 0.005], pre-second BoNT/A 373.3 ± 215.7 mL vs. 656.9 ± 318.7 mL at 1 month [*p* = 0.002] and 500.4 ± 300.1 mL at 6 months [*p* = 0.005]).

No major adverse events occurred during the study. All patients tolerated the procedure well under local or light general anesthesia, and no cases of systemic toxicity or generalized muscle weakness were recorded. Apart from the DM patient, all selected patients were on clean intermittent catheterizations (CICs) before BoNT/A treatment. The DM patient did not require CIC post-treatment.

#### 2.1.1. Microbiological Findings Before and After the First BoNT/A Injection

Before the first BoNT/A injection, urine cultures were positive in 68.2% of patients, indicating a substantial bacterial burden within the study cohort. Of these, 27.3% were symptomatic and received a week’s antibiotic coverage. Only 31.8% of patients had sterile urine cultures prior to treatment (Table 1).

One month following the first BoNT/A administration, a mild decrease in bacterial colonization was observed with positive urine cultures recorded in 14/22 (63.6%) patients; 12 patients (54.5%) remained asymptomatic, and 2 patients (9.1%) developed symptomatic UTIs requiring short courses of oral antibiotics. The clinical presentation of the UTIs was generally mild and easily manageable.

At 6 months, a marked reduction in bacterial colonization was noted: only 35.7% of patients had positive cultures, and just one patient (7.1%) developed a symptomatic UTI. By twelve months, the proportion of patients with positive urine cultures had decreased further to 16.7%, all asymptomatic, while 83.3% of the tested patients demonstrated sterile urine (Table 1).

#### 2.1.2. Microbiological Findings Before and After the Second BoNT/A Injection

Six patients received a second BoNT/A injection approximately one year after the initial treatment. At that time, all six (100%) exhibited positive urine cultures, and two (33.3%) were symptomatic. Three- or seven-day antibiotic regimens were administered as per protocol. One month after the second injection, the proportion of positive urine cultures was reduced by half, with 50% of samples being sterile and the remaining 50% positive but asymptomatic. At six months, although all three patients (100%) with available follow-up cultures had bacterial growth, none experienced urinary symptoms or complications (Table 2).

#### 2.1.3. Trends in Infection Rates and Symptomatic UTI Episodes

**Figure 1:**
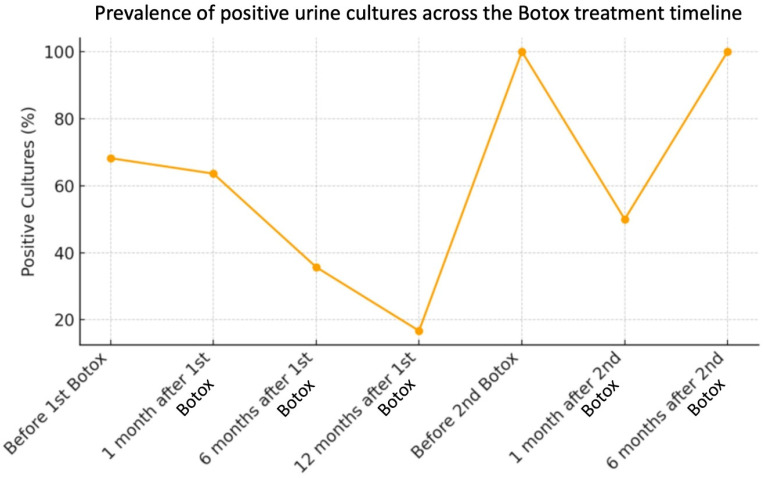
Prevalence of positive urine cultures across the BoNT/A treatment timeline. (Line plot showing a transient rise at 1 month followed by progressive decline through 12 months).

**Figure 2:**
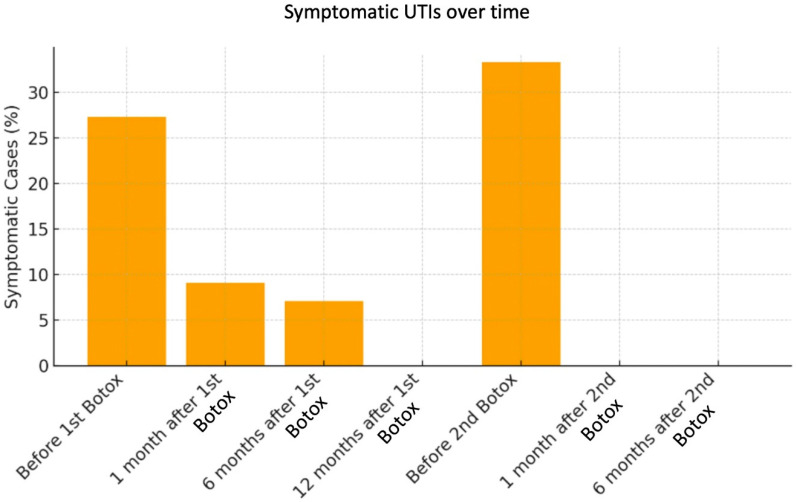
Symptomatic urinary tract infections over time. (Bar plot demonstrating that symptomatic infection remained rare and mild throughout follow-up).

Figure 1 illustrates the overall trend of positive urine cultures across the study period. After a transient rise at one month, there was a consistent and clinically significant decline up to 12 months following the first injection. The proportion of patients with symptomatic infections remained low at all time points, peaking at only 9.1% one month after the first injection (Figure 2).

### 2.2. Evaluation of mRNA Levels of Inflammation and Pathogen Recognition-Associated Factors in Urine as Potential Biomarkers for Bladder BoNT/A Treatment

Patients with an overactive bladder (OAB) have been found to have increased mRNA levels of some urine biomarkers associated with inflammation (such as TNFα, IL2, NGF) [5]. In order to evaluate the effects of BoNT-A bladder injections on additional biomarkers associated with inflammation and urinary infections in patients with OAB, we explored the mRNA levels of TLR2, TLR4, and TLR5 in the urine of patients before and after intradetrusor BoNT/A injections, together with the mRNA levels of inflammatory cytokines, IL1b, IL6, TNFα, and prostaglandin E2 (PGE_2_). Eighteen patients were included who had at least one bladder BoNT/A treatment and had urine specimens adequate for analysis at one month post-treatment (T1), while ten specimens were evaluable for processing at 6 months post-BoNT/A (T2). Another four patients who had a second BoNT/A treatment and had specimens obtained upon the second injection (T3) and one month post-treatment (T4) were also included. In order to define whether there are changes in the mRNA abundance of urine factors involved in pathogen recognition and inflammation, their relative mRNA levels were determined. Towards this goal, the relative mRNA of TLR2, TLR4, TLR5, PGE_2_, IL1β, TNFα, IL6 in urine samples from the patients was evaluated by RT-PCR, before first BoNT/A treatment (T0), one month after first BoNT/A (T1), six months after first BoNT/A treatment (T2), at second BoNT/A treatment (T3), and one month after second BoNT/A treatment (T4).

**Figure 3:**
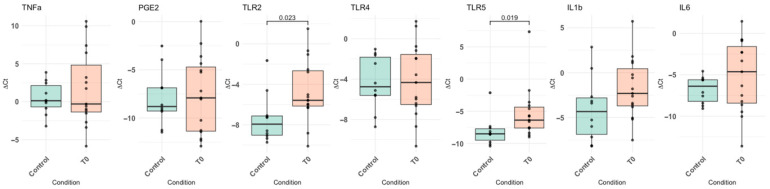
Comparison of the mRNA levels of pathogen recognition and inflammatory factors in urine samples of NDO patients with the control group. Boxplots of the ΔCt values. Statistically significant associations are shown with the level of significance set to *p*-value < 0.05.

In addition, the relative mRNA levels of all genes tested from the 18 patients at baseline were compared to the relative mRNA levels determined in the urine samples of the 10 healthy controls (5 women, 5 men, mean age 39 years). As per Figure 3, at T0 the mRNAs of all factors tested were upregulated in the patients’ samples compared with control samples; however, only TLR2 and TLR5 mRNA increases were statistically significant.

**Figure 4:**
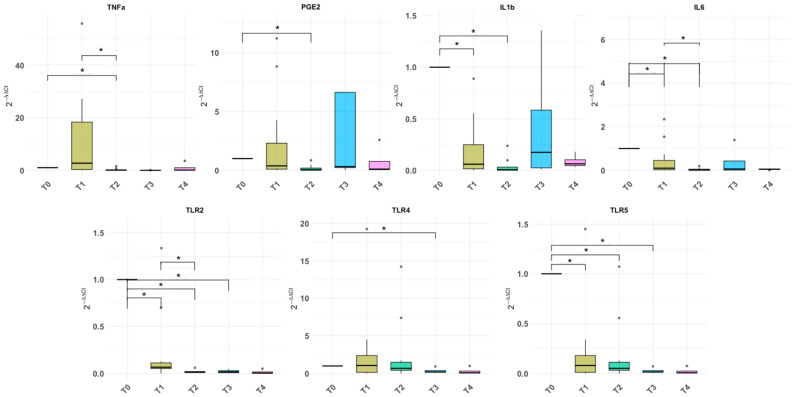
Changes in the mRNA levels of pathogen recognition and inflammatory factors in urine samples of NDO patients before first BoNT/A injection (T0), one month after the 1st BoNT/A injection (T1), six months after 1st BoNT/A injection (T2), at clinical relapse (timepoint of 2nd injection- T3), and at one month after the 2nd BoNT/A injection (T4). The mRNA levels before the first BoNT/A injection (T0) correspond to the mean sample values. Boxplots of the 2^−∆∆CT^ values. Statistically significant associations are shown with an asterisk (*) with the level of significance set to *p*-value < 0.05.

In order to determine whether a single BoNT/A treatment affected the mRNAs under investigation, their levels were compared at T0, T1, and T2. With the exception of TNFα and PGE_2_, all other mRNAs were downregulated after a single BoNT/A treatment (Figure 4). More specifically, TLR2, TLR5, IL1β, and IL6 mRNA levels showed statistically significant progressive reductions at 1 month post-BoNT/A treatment (T1), which were more substantial at 6 months post-treatment (T2). TNFα and PGE_2_ mRNA levels appeared to increase (although not statistically significantly) at 1 month, followed by a dramatic reduction at 6 months. A similar trend was found for TNFα after the second BoNT/A injection. Interestingly, the mRNA levels of PGE_2_, IL-1β, and IL6 increased before the second injection, reflecting the clinical relapse, which was documented by urodynamic investigation in all cases. By contrast, the TNFα, TLR2, TLR4, and TLR5 mRNA levels remained significantly lower than the T0 levels, showing no tendency to mirror the clinical relapse (Figure 4, Table 3).

## 3. Discussion

To our knowledge, this is the first human study measuring the concurrent effect of intradetrusor BoNT/A injections on the mRNA levels of inflammatory and immune-related genes in patients’ urine, also in parallel with changes in the prevalence of UTIs. The findings of the present pilot study suggest a significant reduction in the mRNA levels of inflammatory and immune-related markers following intravesical BoNT/A treatment. More specifically, a progressive downregulation of TLR2, TLR5, IL-1β, and IL-6 urine mRNAs was observed that was sustained at six months. TNFα and PGE_2_ followed a different pattern, with an initial, transient increase at one month after the injection and a dramatic drop at the six months’ follow-up. A similar trend for progressive decline was also noticed in the prevalence of both positive urine cultures and symptomatic UTIs at the same timepoints and additionally at 12 months post-treatment in patients who still had a beneficial response to the BoNT/A treatment. Upon clinical relapse, the mRNA levels of PGE_2_, IL-1β, and IL6 increased in parallel with an increase in the prevalence of UTIs, suggesting an association between bladder inflammation and the resurgence of UTIs, while the levels of pathogen-detecting Toll-like receptors and of TNF-α interestingly did not follow the same trend.

Toll-like receptors (TRLs) recognize pathogen-associated molecular patterns (PAPMs), which are preserved in microorganisms and are necessary for their survival, such as lipopolysaccharides, peptidoglycans, lipoproteins, and lipopolypeptides, as well as the nucleic acids of the viruses [14]. The expression of TRLs in the urothelial cells plays a key role in immune response against UTIs, with TLR2, TLR4, and TLR5 considered to be most important in bacterial infections [15]. In a diabetic type I mouse model, the inane activation of the immune system via TLR4 activation was found to result in inflammation and oxidative stress and to contribute to bladder wall hypertrophy and bladder overactivity [16]. In our study, TLR4 appeared to be the least clinically significant of the TLRs, as its changes post-BoNT/A were non-significant and did not follow the change in the incidence of UTIs. TLR2 and TLR5, on the contrary, demonstrated significant changes post-BoNT/A in parallel with the decrease in UTIs, albeit not at clinical relapse, suggesting possible clinical significance, which warrants further investigation.

The existing body of evidence, regarding the effect of BoNT/A, stems from a diverse pool of studies, including animal and human studies, different cohorts of patients, biomarkers, and methods of measuring them. A study in rats has shown that BoNT/A significantly inhibits the release of substance P and CGRP (calcitonin gene-related peptide) [9]. Regarding human studies, a study in 23 women with interstitial cystitis/bladder pain syndrome (IC/BPS) that assessed the effect of the treatment with bladder biopsies revealed a reduction in Bax and phospho-p38 (p-p38) expression after a single BoNT/A treatment and additional reduction in tryptase, 25-kD synaptosomal-associated protein and apoptotic cell counts, which could imply an anti-inflammatory role of BoNT/A in patients with IC/BPS [14]. Histopathological reduction in VEGF levels has also been observed in IC/BPS patients undergoing BoNT/A treatment with hydrodistention [17].

Similar findings to the present study were reported by a study of 56 patients with non-neurogenic OAB, who had a significant decrease in blood serum PGE_2_ levels after BoNT/A treatment, while the non-responder group demonstrated higher levels of PGE_2_ post-BoNT/A [18]. A study exploring the effect of BoNT/A treatment on nerve growth factor (NGF) in 26 patients with idiopathic OAB also demonstrated a decrease in the levels of NGF in blood serum [19]. The urinary levels of NGF have been assessed by a cross-sectional study in a mixed cohort of 143 patients with idiopathic detrusor overactivity (IDO) and 100 with NDO. The pre-treatment urine NGF levels were higher in the treatment group than in the controls, and the post-treatment urine NGF was lower in the BoNT/A responder group [20]. The role of NGF and PGE_2_ in OAB is well-established, with their levels being higher in the urine of OAB patients than in healthy controls [21,22]. In animal models and in vitro studies, intravesical PGE_2_ appears to induce OAB symptoms and detrusor overactivity [23,24]. A study of 30 women with OAB who received anticholinergic treatment reported a reduction in urine NGF levels but not in PGE_2_, even in the responder group [21]. This is an interesting observation, with the differential effect of anticholinergics and BoNT/A on NGF and PGE_2_ potentially being attributed to their distinct mechanisms of action. Anticholinergics decrease bladder overactivity and the afferent signaling via blockade of muscarinic receptors. The reduction of detrusor overactivity and, consequently, of the mechanical stretching of the bladder could reduce the NGF levels, as has been shown in in vitro studies [25,26]. PGE_2_ on the other hand is primarily synthesized via the cyclooxygenase (COX) pathway, as part of the local inflammatory response [27]. This observation further supports the theory that BoNT/A exerts anti-inflammatory action, which is the result not only of the decrease in detrusor overactivity but possibly of a broader action on the urothelium.

In our study cohort, the overall incidence of symptomatic UTIs remained low throughout follow-up, and no hospitalization or intravenous antibiotic therapy was required. The progressive decline noted in UTIs is in accord with earlier observational studies. The overall rate of positive urine cultures showed a similar trend, implying a decrease in bacterial colonization. From a clinical perspective, it could be argued that repeated bladder emptying and improved detrusor function following BoNT/A treatment may contribute to a more favorable urological microbiome and decreased infection risk over time. Moreover, in light of the biomarker results, our findings suggest that intradetrusor BoNT/A injections may actually positively and significantly affect the background associated with the incidence of UTIs. Further to the post-BoNT/A reductions in the mRNA levels of TLRs, which could be secondary to the reduced incidence of UTIs, the reduction in urine mRNA levels of inflammatory cytokines IL1β, IL6, and of PGE_2_ suggests a reduction in the inflammatory background in the patients’ bladders but may also suggest a lessened immune response, i.e., a reduced incidence of UTIs. In addition, the repeat injection findings suggest that, although bacterial colonization may persist in patients requiring repeat injections, BoNT/A therapy does not appear to precipitate symptomatic infection. The pattern indicates that bacterial persistence may reflect underlying bladder dysfunction rather than a treatment-induced effect.

Study limitations: The sample size is small, and no a priori power calculations were performed as this was an exploratory study. A significant follow-up attrition was noted at the 12-month follow-up, as a number of patients had clinical relapse before this timepoint. As the biomarker study did not extend to the 12-month clinical follow-up, attrition at 12 months may have affected the robustness of the UTI data but not the robustness of the biomarker data. Further limitations may lie with the control group, which differs substantially from the patient cohort in disease status, comorbidities, and medication exposure, while it was also not possible to achieve age- and sex-matching due to the different group sizes. Ideally, as a non-DO/non-OAB neurogenic control group is difficult to create when recruiting from a neurourology outpatient clinic, a second control group with non-neurogenic OAB could be added for more meaningful comparisons in a novel, more specifically designed study. The use of antibiotics, which could have altered UTI incidence, urinary microbiota, and inflammatory and immune genes expression, may constitute another limitation to the interpretation of our results. Even so, however, as antibiotics were routinely used to treat clinical UTIs and as prophylaxis before each BoNT/A injection but not routinely before follow-up urodynamics, both the higher rates of UTIs and the higher mRNA levels of all biomarkers before BoNT/A treatment as opposed to post-BoNT/A timepoints suggests that, whilst an effect of antibiotics on our results cannot be excluded, the findings should be mainly attributed to the effect of BoNT/A.

Finally, RNA detection in the urine has been demonstrated to be a reproducible noninvasive method for evaluation of biomarkers for prognosis and diagnosis [28]. The source of RNA in the urine was not investigated in our study, but previous publications have indicated that the origin of urine RNA may be from exfoliated cells that have been shed into the urine from the urinary tract, immune cells [29], or exosomes [30]. The origin of RNAs under investigation in our study cannot be bacterial, because the primers used for RT-PCR exclusively amplify the human genes of interest.

## 4. Materials and Methods

### 4.1. Study Design

This pilot, prospective study was approved by the Hospital Scientific Board (ref. No.: 89/22 January 2021), and all participants were recruited following written informed consent. Patients with NDO-associated incontinence refractory to oral pharmacotherapy who received bladder BoNT/A injections were recruited. As per routine protocol approved by the Hospital Scientific Board, all patients were subjected to urodynamic investigation pre-treatment with BoNT/A (baseline), at 4–6 weeks, and at 6 and 12 months post-treatment. Response to treatment was determined by significant improvements in maximum cystometric capacity (MCC) in conjunction with significant improvement in incontinence. Complete response was defined as 90–100% improvement in incontinence with pad use discontinuation or use of one safety pad per day as reported by the patient and at least 50% improvement in MCC. Patients were considered eligible for re-injection when improvement in incontinence dropped below 50%, demonstrated by a return of urodynamically proven incontinence and decrease in MCC close to baseline levels.

Urine specimens for the study of biomarkers were obtained at baseline during the BoNT/A treatment and upon routine urodynamic follow-up visits at 4–6 weeks and at 6 months post-treatment. Urine specimens were also obtained from 10 adult healthy volunteers with no OAB symptoms and served as the control group in the biomarker study.

All patients were screened for UTIs 7–10 days before the BoNT/A injection. If symptomatic with a positive culture, they received appropriate antibiotic medication for 7 days, followed by the BoNT/A treatment. If asymptomatic but with a positive culture, they received a 3-day antibiotic scheme based on the urine culture, as per the approved injection protocol. Finally, patients with clean urine cultures received antibiotics one day before the BoNT/A treatment. Additionally, all patients received oral antibiotics on the day of the injection and for 3–5 days afterwards. To study the effect of BoNT/A on UTIs, urine specimens for culture were also obtained during urodynamic follow-up timepoints, and the findings were compared with the pre-treatment results.

### 4.2. Study Outcomes

The primary outcome was the change in the mRNA levels of TLR2, TLR4, and TLR5 in the urine of patients at 1 and 6 months post-treatment compared with baseline. The secondary outcomes included the following:

- The change in the mRNA levels of IL1b, IL6, TNFα, and PGE_2_ in the urine of patients at 1 and 6 months post-treatment compared with baseline.
- The comparison of the mRNA levels of TLR2, TLR4, TLR5, IL1b, IL6, TNFα, and PGE_2_ in the urine of patients between the active treatment arm at baseline and the control group.
- The change in the percentage of patients with UTI at 1, 6, and 12 months post-treatment compared with baseline.
- The change in all studied mRNA levels of interest in patients who received a 2nd BoNT/A injection at one month after the 2nd injection. Also, comparison of baseline mRNA levels of all genes between the 1st and 2nd injections.

### 4.3. Laboratory Techniques

Specimens were preserved using the Urine Preservative Single Dose^®^ by Norgen Biotek Corpοration, Thorold, ON, Canada, and stored at room temperature. This prevents the growth of Gram-negative, Gram-positive bacteria and fungi and also inactivates viruses. Additionally, it is suitable for preserving samples for more than 2 years at room temperature without detectable degradation of DNA, RNA, or proteins. RNA extraction, cDNA synthesis, and real-time PCR (RT- PCR): Extraction of mRNA from urine was performed using Urine Exfoliated Cell RNA Purification Kit (Norgen Biotek Corp, Thorold, ON, Canada) according to the manufacturer’s instructions. This kit provides a rapid method for the isolation and purification of total RNA from exfoliated cells that have been shed into the urine from the urinary tract.

Briefly, 1 μg of total RNA was transcribed to cDNA using reverse transcriptase (Luna Script RT Super Mix—New England Biolabs, Cambridge, UK) according to the manufacturer’s instructions. Analysis of cDNA samples by real-time PCR was performed using the Applied Biosystems StepOne system v2.3 and Luna Universal qPCR Master Mix (New England Biolabs, Cambridge, UK) according to the manufacturer’s instructions. The PCR program included 1 cycle at 95 °C for 1 min, 43 cycles at 95 °C for 15 s, 60 °C for 30 s, followed by melting curve analysis up to 95 °C. The threshold cycle (Ct) value for each gene was normalized to the Ct value for housekeeping gene GAPDH. Relative expression levels were determined by the 2^−∆∆CT^ algorithm. The sequences of the primers used for RT-PCR are provided in Table 4.

### 4.4. Statistical Analysis

As this study was an exploratory one, no a priori power calculation was performed. The paired *t*-test was used for comparisons between baseline and follow-up MCC values. The 2^−∆∆CT^ algorithm was used to determine the relative changes in mRNA levels. The level of significance was set to *p*-value < 0.05. The Wilcoxon Rank Sum test and the visualizations of the ΔCt and ΔΔCt values were implemented in R v.4.3.2.

## 5. Conclusions

In our cohort of patients with NDO-associated incontinence, intradetrusor BoNT/A injections achieved significant decreases in the urine mRNA levels of Toll-like receptors associated with the detection of urinary tract pathogens, inflammatory cytokines, and prostaglandin PGE2 associated with the mediation of immune response and inflammation. Changes in urine biomarkers appeared to happen in parallel with decreases in both the incidence of symptomatic UTIs but also collective bacterial colonization reflected in reduced rate of positive urine cultures, either asymptomatic or symptomatic. As the biomarkers of inflammation and immune response, but not the TLRs, increased at the time of clinical relapse and pending larger studies, the studied genes PGE2, IL-1β, and IL6 may be further explored as potential biomarkers for inflammation/immune response and UTIs in the neurogenic population.

## Figures and Tables

**Table 1:** Urine culture results before and after the first BoNT/A injection (n = 22).

Timepoint	Negative Culture (%)	Positive Culture, No Symptoms (%)	Positive Culture, Symptomatic (%)	Any Positive Culture (%)
Baseline	31.8	40.9	27.3	68.2
1 month after 1st injection	36.4	54.5	9.1	63.6
6 months after 1st injection	64.3	28.6	7.1	35.7
12 months after 1st injection	83.3	16.7	0.0	16.7

**Table 2:** Urine culture results before and after the second BoNT/A injection (n = 6).

Timepoint	Negative Culture (%)	Positive Culture, No Symptoms (%)	Positive Culture, Symptomatic (%)	Any Positive Culture (%)
Before 2nd injection	0	66.7	33.3	100
1 month after 2nd injection	50.0	50.0	0.0	50.0
6 months after 2nd injection	0	100.0	0.0	100.0

**Table 3:** Statistical significance of the changes in the mRNA levels of the potential biomarkers at the various timepoints after the 1st and 2nd BoNT/A injections. Absence of *p*-values signifies lack of statistical significance at the respective timepoints.

*p*-Values	T0–T1	T0–T2	T1–T2	T0–T3
TNFα		0.005	0.005	
PGE2		0.00064		
IL1b	0.00048	0.00048		
IL6	0.012	0.00064	0.039	
TLR2	0.002	0.00064	0.012	0.002
TLR4				0.006
TLR5	0.002	0.004		0.002

**Table 4:** The sequences of the primers used for RT-PCR.

**TNFα** F: 5′- GCT GCA CTT TGG AGT GAT CG-3′ R: 5′- GCT TGA GGG TTT GCT ACA ACA-3′
**TLR2** F: 5′- CTT CAC TCA GGA GCA GCA AGC A -3′ R: 5′- ACA CCA GTG CTG TCC TGT GAC A-3′
**TLR4** F: 5′- CCC TGA GGC ATT TAG GCA GCT A -3′ R: 5′- AGG TAG AGA GGT GGC TTA GGC T-3′
**TLR5** F: 5′- CCT TAC AGC GAA CCT CAT CCA C -3′ R: 5′- TCC ACT ACA GGA GGA GAA GCG A-3′
**PGE_2_** F: 5′- GCT CCT TGC CTT TCA CGA TTT-3′ R: 5′- AGG ATG GCA AAG ACC CAA GG -3′
**IL6** F: 5′- AGC CCT GAG AAA GGA GAC ATG TAA-3′ R: 5′- GAA TGA GGA CAC ACC CAC CTT-3′
**IL1b** F: 5′- GCT GCT CTG GGA TTC TCT TCA-3′ R: 5′- TCC TGG AAG GAG CAC TTC ATC T -3′

## Data Availability

Both clinical and laboratory research data can be available upon request to the authors.

## Abbreviations

The following abbreviations are used in this manuscript:

NDO	neurogenic detrusor overactivity
UTIs	urinary tract infections
OAB	overactive bladder
BoNT/A	botulinum neurotoxin type A
TLRs	Toll-like receptors
PGE_2_	prostaglandin E_2_
TNFα	tumor necrosis factor alpha
IL	interleukin
CGRP	calcitonin gene-related peptide
IC/BPS	interstitial cystitis/bladder pain syndrome
NGF	nerve growth factor
RT-PCR	real-time polymerase chain reaction
IDO	idiopathic detrusor overactivity
